# Targeting the immunoproteasome in hypothalamic neurons as a novel therapeutic strategy for high-fat diet-induced obesity and metabolic dysregulation

**DOI:** 10.1186/s12974-024-03154-z

**Published:** 2024-08-02

**Authors:** Nicolás Albornoz, Javiera Álvarez-Indo, Adely de la Peña, Eloisa Arias-Muñoz, Alanis Coca, Fabián Segovia-Miranda, Bredford Kerr, Mauricio Budini, Alfredo Criollo, María A. García-Robles, Eugenia Morselli, Andrea Soza, Patricia V. Burgos

**Affiliations:** 1 Centro de Biología Celular y Biomedicina, Facultad de Medicina y Ciencia, Universidad San Sebastián, Santiago, Chile; 2 Departamento de Biología Celular, Facultad de Ciencias Biológicas, Universidad de Concepción, Concepción, Chile; 3 Laboratory of Molecular and Cellular Pathology, Institute in Dentistry Sciences, Dentistry Faculty, University of Chile, Santiago, Chile; 4 Cell and Molecular Biology Laboratory, Institute in Dentistry Sciences, Dentistry Faculty, Universidad de Chile, Santiago, Chile; 5 Advanced Center for Chronic Diseases (ACCDiS), Faculty of Chemical and Pharmaceutical Sciences, Faculty of Medicine, Universidad de Chile, Santiago, Chile; 6 Department of Basic Sciences, Faculty of Medicine and Sciences, Universidad San Sebastián, Santiago, Chile; 7 Centro Basal Ciencia & Vida, Universidad San Sebastián, Santiago, Chile

**Keywords:** Obesity, Immunoproteasome, Hypothalamus, Neurons, Insulin-glucose axis, Autophagy, Mitochondrial function, Metabolic disturbances, Redox biology

## Abstract

**Objective:**

Obesity represents a significant global health challenge characterized by chronic low-grade inflammation and metabolic dysregulation. The hypothalamus, a key regulator of energy homeostasis, is particularly susceptible to obesity’s deleterious effects. This study investigated the role of the immunoproteasome, a specialized proteasomal complex implicated in inflammation and cellular homeostasis, during metabolic diseases.

**Methods:**

The levels of the immunoproteasome β5i subunit were analyzed by immunostaining, western blotting, and proteasome activity assay in mice fed with either a high-fat diet (HFD) or a regular diet (CHOW). We also characterized the impact of autophagy inhibition on the levels of the immunoproteasome β5i subunit and the activation of the AKT pathway. Finally, through confocal microscopy, we analyzed the contribution of β5i subunit inhibition on mitochondrial function by flow cytometry and mitophagy assay.

**Results:**

Using an HFD-fed obese mouse model, we found increased immunoproteasome levels in hypothalamic POMC neurons. Furthermore, we observed that palmitic acid (PA), a major component of saturated fats found in HFD, increased the levels of the β5i subunit of the immunoproteasome in hypothalamic neuronal cells. Notably, the increase in immunoproteasome expression was associated with decreased autophagy, a critical cellular process in maintaining homeostasis and suppressing inflammation. Functionally, PA disrupted the insulin-glucose axis, leading to reduced AKT phosphorylation and increased intracellular glucose levels in response to insulin due to the upregulation of the immunoproteasome. Mechanistically, we identified that the protein PTEN, a key regulator of insulin signaling, was reduced in an immunoproteasome-dependent manner. To further investigate the potential therapeutic implications of these findings, we used ONX-0914, a specific immunoproteasome inhibitor. We demonstrated that this inhibitor prevents PA-induced insulin-glucose axis imbalance. Given the interplay between mitochondrial dysfunction and metabolic disturbances, we explored the impact of ONX-0914 on mitochondrial function. Notably, ONX-0914 preserved mitochondrial membrane potential and attenuated mitochondrial ROS production in the presence of PA. Moreover, we found that ONX-0914 reduced mitophagy in the presence of PA.

**Conclusions:**

Our findings strongly support the pathogenic involvement of the immunoproteasome in hypothalamic neurons in the context of HFD-induced obesity and metabolic disturbances. Targeting the immunoproteasome highlights a promising therapeutic strategy to mitigate the detrimental effects of obesity on the insulin-glucose axis and cellular homeostasis. This study provides valuable insights into the mechanisms driving obesity-related metabolic diseases and offers potential avenues for developing novel therapeutic interventions.

**Supplementary Information:**

The online version contains supplementary material available at 10.1186/s12974-024-03154-z.

## Introduction

Obesity, acknowledged as a global epidemic, is significantly promoted by unbalanced diets high in saturated fats such as palmitic acid (PA) [1]. Chronic consumption of such HFD has been associated with increased metabolic diseases, including an imbalance in the insulin-glucose signaling axis, leading to insulin resistance [2, 3]. In mice, chronic consumption of these diets leads to the accumulation of PA in the brain, specifically the hypothalamus, the critical region responsible for maintaining energy homeostasis and body metabolism [4].

Macroautophagy, hereafter autophagy, a crucial cellular homeostatic mechanism, is inhibited in the hypothalamus by this type of diet [5, 6]. Indeed, in mice exposed to a 16-week HFD, the protein levels of Beclin1 and LC3-II decreased, while the protein content of p62/SQSTM1 increased when compared to lean mice fed with a regular diet [6]. The autophagy flux directly quantifies the amount of autophagy-dependent degradation of cellular components such as Beclin1, p62/SQSTM1, LC3 ratio, among others. In hypothalamic neurons, a decrease in autophagy inhibits insulin signaling and insulin-dependent glucose uptake [7]. Consistently, inhibition of autophagy in the hypothalamus increases blood glucose and insulin levels and drives chronic low-grade inflammation via IKKβ/NF-κB activation [5].

One of the major functions of autophagy is to counteract the inflammatory responses [8]. Persistent inflammation, broadly identified in obesity, disrupts the hypothalamic function in energy balance, leading to accelerated development of obesity and its comorbidities [9]. The energy balance is finely regulated to maintain an equilibrium between energy intake (food we eat) and energy expenditure (calories we burn). It acts as a control center, constantly monitoring and integrating signals from various body parts to regulate hunger, satiety, and metabolism.

The molecular components activated in response to a decrease in autophagic flux that could lead to an imbalance in the insulin-glucose axis remain largely unknown, a question of significant interest in the field of biomedicine. One possible candidate is the immunoproteasome, prevalent in inflammatory scenarios [10, 11]. The immunoproteasome is a proteolytic protein complex ubiquitously expressed during inflammation where catalytic subunits of the standard proteasome, β1, β2, and β5, are replaced by the inducible subunits; β1i/LMP2, β2i/MECL-1, and β5i/LMP7, respectively, forming an alternative isoform [12, 13]. Immunoproteasomes are capped at one (26 S) or both ends (30 S) by the 19 S regulatory particle that captures and prepares appropriate substrates for breakdown [13]. Since the ratio of immunoproteasome, 26 S immunoproteasome or 30 S immunoproteasome varies across different cellular conditions, a dynamic equilibrium between the two species may be part of an adaptive response to cellular needs [13]. Deleting the immunoproteasome’s β5i subunit in mice reduces the obesogenic phenotype and insulin resistance in response to an HFD [14]. Yet, whether HFD leads to an increase in the immunoproteasome in the hypothalamus or if this complex could be linked to an imbalance in the insulin-glucose axis is unknown. Furthermore, the potential contribution of the immunoproteasome to cellular metabolism, particularly in hypothalamic neurons, remains entirely unexplored.

## Materials and methods

### Chemical reagents

Products were purchased from several suppliers. From Sigma-Aldrich (St. Louis, MO, USA), we procured Amersham™ Protran® Nitrocellulose (cat#GE1060002), Palmitic Acid (PA, cat#P0500), Stearic Acid (SA, cat#S4751), Type A Porcine Skin Gelatin (cat#G-2500), 2-b-Mercaptoethanol (cat#M3148), and protease inhibitor “cocktail” (cat#P8340). Bovine Serum Albumin (BSA, cat#BSA-05) for antibody dilution was obtained from Rockland Immunochemicals (Pottstown, PA, USA), while fatty acid-free BSA (cat#152,401), used as a treatment, came from MP Biomedicals (Santa Ana, CA, USA). ONX-0914 (cat#HY-13,207) was procured from MedChemExpress (Monmouth Junction, NJ, USA), and insulin (humulin R 100UI/ml F-A) was acquired from Cruz Verde (Santiago, Chile). Merck Millipore (Burlington, MA, USA) supplied us with Methanol (cat#1,060,092,500), Acetic Acid (cat#100064.1000), and HEPES-free acid (cat#391,340). ThermoFisher Scientific (Waltham, MA, USA) provided numerous items including the BCA Kit (cat#23,225), DMEM (cat#D5030-10 L), and many other biological reagents. From Winkler (Santiago, Chile), we purchased Acrylamide: Bisacrylamide 29:1 30% (cat#BM0100), Bromophenol Blue (cat#A2-0395), and several other chemicals. SDS (cat#C15081006) and Triton X-100 (cat#T8655) were purchased from USBiological Life Science (Salem, Massachusetts, USA). Our lab also used Fluoromount-G (cat#17984-25) and 16% Paraformaldehyde molecular grade solution (PFA cat#15,710) from Electron Microscopy Science (Hatfield, PA 19,440, USA), and Glycine (cat#FER00G500G) and Tris Base (cat#FER00B500) from Fermelo Biotec (Santiago, Chile). The AccuRuler RGB Plus Ladder Protein (cat#02102 − 250) was purchased from MaestroGen (Hsinchu City, Taiwan, China). Other critical reagents include Ammonium persulfate (APS, cat#0486 − 256) from Avantor-VWR (Radnor, PA, USA), Fetal bovine serum (FBS, cat#04-127-1 A) from BI Biological Industries/Sartorius (Aubagne, France), Phosphate-buffered saline (PBS, cat#46-013-CM) from Corning Inc. (Glendale, Arizona, USA), Tween-20 (cat#SC29113B) from ChemCruz™ Biochemicals-Santa Cruz Biotechnology (Dallas, TX, USA), and the fluorogenic peptide Ac-ANW-AMC specific for β5i (Cat#26,640) from Cayman Chemical.

### Antibodies

Monoclonal antibodies used in this study were sourced from three companies. From Abnova, we used Mouse p62/SQSTM1 (Clone 2C11, cat#H00008878-M01, Dilution 1:5000 in 3% BSA and 1:300 in 0.2% Gelatin). All primary and secondary antibodies were diluted in 3% BSA. From Santa Cruz Biotechnology, we utilized Mouse BECN1 (cat#sc-11,427, Dilution 1:1000), Mouse PTEN (cat#sc-7974, Dilution 1:1000), Mouse β5 (cat#sc-393,931, Dilution 1:1000), Mouse GLUT4 (cat#sc-53,566, Dilution 1:1000), and Mouse β-Actin (cat#sc-47,778, Dilution 1:2000). Lastly, from Abcam, we used Mouse Hexokinase-II (Clone 3D3, cat#ab104836, Dilution 1:1000). For polyclonal antibodies, we used four suppliers. From Cell Signaling Technology, we used Rabbit p-AKT (Ser473) (cat#9271, Dilution 1:2000), Rabbit AKT (cat#9272, Dilution 1:2000), and Rabbit TOM20 (Clone D8T4N, cat#42,406, Dilution 1:200 in 0.2% Gelatin). We also utilized Rabbit FIP200 from ProteinTech (cat#172,501-AP, Dilution 1:1000), Rabbit β5i from Abcam (cat#Ab3329, Dilution 1:1000), Rabbit Glucose 6 Phosphate Dehydrogenase (Clone EPR6292, cat#ab133525, Dilution 1:1000), and Rabbit Lactate Dehydrogenase (Clone EPR1564, cat#ab101562, Dilution 1:1000). Lastly, Rat LAMP1 was sourced from BDPharmingen (Clone 1D4B, cat#553,792, Dilution 1:300 in 0.2% Gelatin).

### Use of mice and HFD diet

Four-week-old male C57BL/6 mice were obtained from the animal facility of Fundación Ciencia & Vida. Then, mice were housed for 8 weeks in the mouse room of the CEBICEM-USS. Housing conditions included an average temperature of 21 °C, daily 12-h light-dark cycles, and ad libitum access to water. Four-week-old male mice were fed either a CHOW diet (cat # 0001495, Lab Diet Inc.) or HFD (cat# D12492, Research Diets, Inc.) for 8 weeks.

### Cell culture and treatments with fatty acids, drugs, insulin, or siRNAs

Human HeLa WT cells (ATCC # CCL-2), HeLa ATG9aKO cells (kindly donated by Dr. Juan Bonifacino, NIH and referred to in [15]), human HeLa ATG8KO cells (kindly donated by Dr. Maho Hamasaki and referred to in [16]), mouse embryonic hypothalamic N43/5 cells (Cellutions Biosystems, code #CLU127), and adult mouse hypothalamic CLU177 cells (Cellutions Biosystems, code #mHypoA-2/12- CLU177) were used. All cell lines were cultured and grown in DMEM culture medium (cat#11965092, ThermoFisher Inc), supplemented with 10% v/v fetal bovine serum (FBS), 100 U/mL penicillin, and streptomycin at 37°C with 5% CO_2_. Cells were cultured on glass coverslips at 15,000 cells per well. In 6-well plates, 200,000 cells were seeded per well. Treatments were performed no earlier than the third day after seeding. Treatment with fatty acids: N43/5 and CLU177 cells were pretreated with DMEM (1 g/L glucose; #A1443001, ThermoFisher Inc) supplemented with 2% FBS for 24 h. Then, they were incubated with 100 µM PA, or SA conjugated to BSA 100 µM for 6 and 24 h. BSA 100 µM alone at the same concentration was used as a control. Drug treatment: CLU177 cells were assessed with the commercial inhibitor ONX-0914 (10 nM prepared in DMSO) to study the effect of immunoproteasome inhibition in response to fatty acids. DMSO was used as a vehicle control. Insulin treatment: CLU177 cells were pretreated with DMEM 1 g/L glucose supplemented with 2% FBS for 24 h. Subsequently, the cells were incubated with commercial insulin (10 nM) for 5, 15, or 30 min, diluted in PBS 1X. siRNA treatment: N43/5 or CLU177 cells were cultured in 6-well plates and transfected with siRNAs when the cells reached 20% confluence. siRNAs for murine Beclin1 (5´-cugagaaugaaugucgaa-3´, Sigma-Aldrich), for FIP200 (5’- GGAGAUUUGGUACUCAUCAUCA-3’, DNA Technologies IDT), or for β5i (5´-AGG AAAGGAAUGUUCAAAUUG-3´, Horizon Discovery Ltd) were used. Each of the siRNAs was prepared at a concentration of 100 µM and used at a concentration of 10 nM. According to the manufacturer’s instructions, the transfection was performed using Lipofectamine RNAiMAX® reagent (Invitrogen, Carlsbad, CA, USA). As a negative control, cells were incubated with siControl (5’- UUCUCCGAACGUGUCACGU-3’, DNA Technologies IDT). After 72 h, the cells were processed for protein extraction using RIPA buffer (50 mM Tris-HCl, 150 mM NaCl, 5 mM EDTA, 1% NP40, 1% sodium deoxycholate, 0.1% SDS, pH 7.4).

### Tissue immunofluorescence

**Fig. 1:**
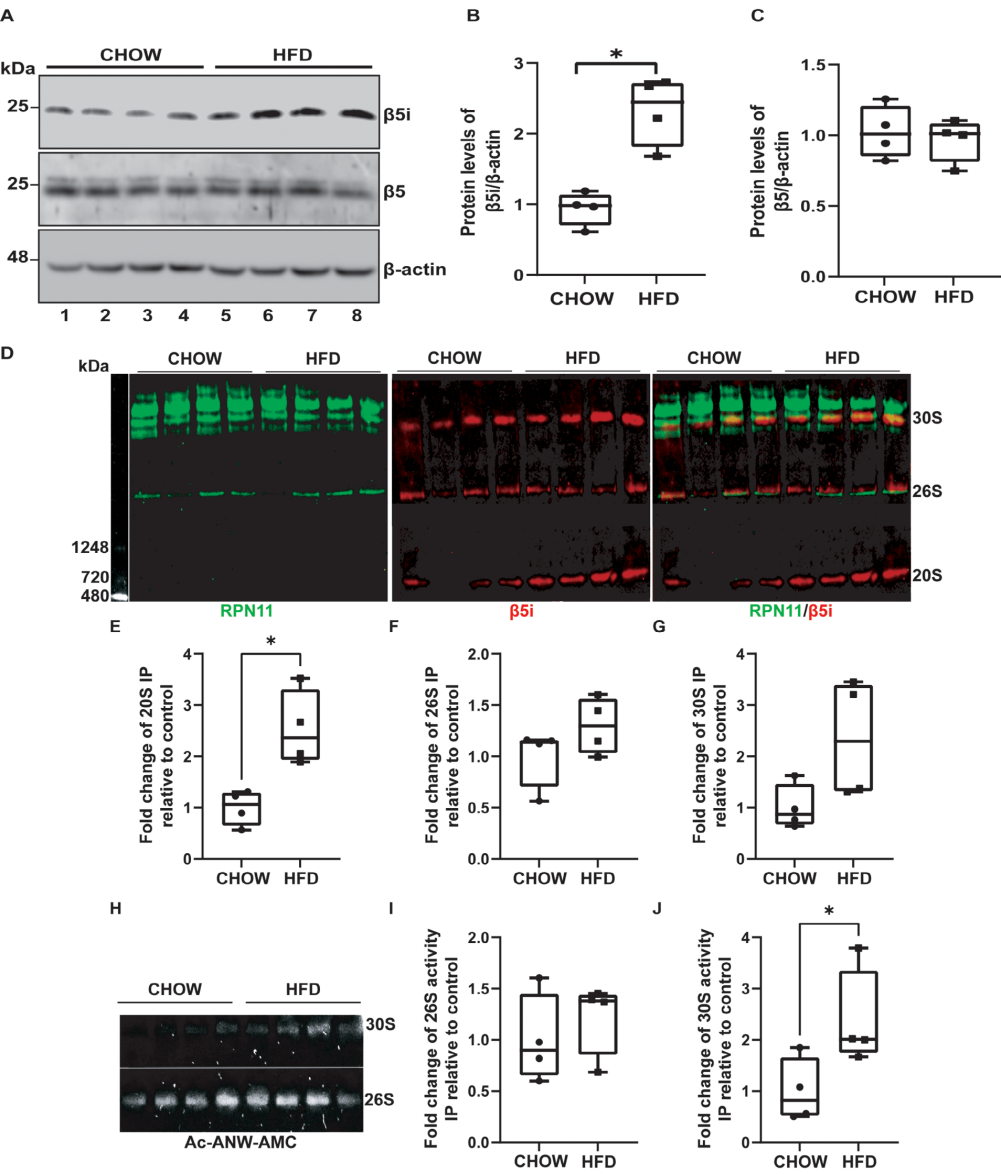
Consumption of an HFD increases the levels of immunoproteasome in hypothalamic astrocytes and POMC neurons (**A**) Protein extracts from hypothalamic lysates of male mice fed with chow or HFD for 16 weeks were analyzed by western blot to evaluate the total levels of β5i and β5 subunits. β-actin was used as internal control. Quantification of β5i (**B**) and β5 (**C**) levels normalized to β-actin. (**D**) Representative native western blot showing the subunits RPN-11 and β5i levels incorporated into the 20 S, 26 S, and 30 S of the immunoproteasome. The RPN11 subunit of the 19 S regulatory particle (green) and β5i subunit of the 20 S complex (red) were analyzed from hypothalamic lysates of male mice fed either with chow or HFD for 16 weeks. Quantification of the β5i levels incorporated to the 20 S (**E**), 26 S (**F**), and 30 S (**G**) complexes is shown. (**H**) Activity of the β5i subunit incorporated into the 26–30 S complex was measured in native gels using the fluorescent substrate Ac-ANW-AMC in hypothalamic lysates from male mice fed either with chow or HFD diet for 16 weeks. Quantification of the activity of the 26 S and 30 S complexes is shown in (**I**) and (**J**), respectively. Data are represented as mean ± SEM. Analysis: Mann-Whitney test. *p < 0.05, *n* = 4

C57BL/6J 16-week-old-Mice or POMC-eGFP mice were anesthetized intraperitoneally with Ketamine/Xylazine (90 mg/kg/10 mg/kg) and vascular perfused first with 50 mL of PBS and second with 50 mL of 4% PFA-0.1% Tween. Subsequently, brain tissue embedded in agarose was cut at 100 μm using a vibratome (Leica, model VT1000 S), following the protocol previously published (Morales-Navarrete et al., 2019). In addition, 50 μm sections were alternatively made using the cryostat (Leica, model 1900-1-1). Then, the sections were processed using an immunofluorescence protocol with the floating section technique (Potts et al., 2020). The agarose was removed with a 0.5% Triton X-100 solution in PBS for 1 h for those sections embedded in agarose. The sections obtained by the cryostat were blocked with 3% BSA in PBS for 1 h. Subsequently, the immunofluorescence protocol proceeded without changes. Primary antibodies were diluted in buffer TX (0.2% gelatin, 300 mM NaCl, 0.3% Triton X-100 in PBS 1X), and a volume of 1:150 µL was incubated with the section for 2 days at 4 °C in a humid chamber. The primary antibodies used were β5i (Abcam, Ab3329), chicken anti-GFP (Synaptic system, # 132,006), and GFAP (Santa Cruz Biotechnology, sc-33,673). Then, 5 washes of 15 min each were performed with PBS-Tween 0.3%. A 200 µL of secondary antibodies diluted 1:1000 in buffer TX coupled to Alexa Fluor® (Life Technologies) was incubated with the sections for 2 days at 4 °C in a humid chamber. Nuclei were co-stained with DAPI (1:10,000) and added to the secondary antibody dilution. Then, the samples were washed 6 times for 5 min in Tris-phosphate buffer (pH 7.8) and mounted with a fluorescence mounting medium (Dako Cytomation, Campintene, CA, USA). Images of the hypothalamus slices were obtained by confocal spectral microscopy (Carl Zeiss, LSM780, Jena, Germany) at ×40 magnification (bidirectional X, speed 600 Hz, pinhole 1.00 AU) and processed using ImageJ software (National Institutes of Health, Bethesda, MD, USA). Images Fig. 1-B and D are two overlapping and correlative images. The images of the tissue samples obtained with the Zeiss LSM780 confocal microscope were separated into three channels. One to observe POMC-GFP neurons, astrocytes (GFAP) coupled with secondary antibody Donkey anti-Mouse IgG H + L, Alexa-488 (#A-21,202, ThermoFisher Inc), immunoproteasome (β5i) coupled with secondary antibody Donkey anti-Rabbit IgG H + L, Alexa-647 (#A-31,573, ThermoFisher Inc) or alternatively nucleus (DAPI). Using ImageJ/Fiji, the AutoNeuriteJ plugin (https://github.com/Grenoble-Institute-Neurosciences/AutoNeuriteJ) was used to generate superimposed images (neurons with nuclei or astrocytes with nuclei). Subsequently, a third overlay is performed with the immunoproteasome (β5i) channel, quantifying the fluorescence intensity, as detailed in [17].

### Indirect immunofluorescence in cell lines

Cells grown on coverslips were washed with 1X PBS and fixed in 4% (v/v) paraformaldehyde for 20 min at room temperature. After fixation, cells were washed in 1X PBS and permeabilized with 0.2% (v/v) Triton X-100 in 1X PBS for 10 min at room temperature. Subsequently, 0.2% pig gelatin in 1X PBS was added for 15 min. Next, cells were incubated with the primary antibodies TOM20, LAMP1, and p62/SQSTM1 for 1 h at 37 °C. Then, coverslips were washed in 1X PBS and incubated with the corresponding secondary antibodies conjugated with Donkey anti-Mouse IgG H + L Alexa-488 (cat#A-21,202, ThermoFisher Inc), Donkey anti-Rat IgG H + L Alexa-594 (cat#A482711, ThermoFisher Inc), and Donkey anti-Rat IgG H + L Alexa-647 (#A32795, ThermoFisher Inc), respectively, at a concentration of 1:1000 in 0.2% pig gelatin in 1X PBS for 30 min at 37ºC. Finally, cells were washed with 1X PBS and incubated for 10 min at room temperature with DAPI (1:10,000). After the final wash, coverslips were mounted on glass slides using Fluoromount-G (Electron Microscopy Science, USA). For quantification of fluorescent signals, 16-bit images were acquired under identical settings avoiding signal saturation and corrected for background signal on each image. The corrected fluorescent signal in each cell of each image was used in Image J (version 1.44o; Wayne Rasband, NIH, http://imagej.nih.gov) to determine the total integrated pixel intensity per cell area. Colocalization analyses were performed with sets of immunofluorescence images of the same cells for each marker. Quantification was performed with the software Image J software version 1.44o(Wayne Rasband, NIH, http://imagej.nih.gov) and the plugin “colocalization threshold” to determine the Manders’ coefficients for each channel. Scores are calculated for pixels above an automatically determined threshold for both channels, according to the algorithm described by [18].

### Live cell imaging in cell lines

HEK293T cells were seeded into 6-well plated at 1 × 10^5^ cells per well in DMEM supplemented with 10% FBS for lentiviral production. A transfection mix contained 1 µg of the plasmid mt-Keima (kindly donated by Dr. Thomas Mund), 0.5 µg of plasmids viral capsid (VSV-G) and retroviral gag/pol (OPG), and 0.1 mg/ml of Polyethyleneimine (PEI) was incubated for 20 min at room temperature before adding to the cells. The virus-containing supernatants were collected after 48 h of incubation at 37 °C and centrifugated at 8000 x g for 3 min at room temperature. Viral supernatants were titrated in 500 µl of DMEM containing 10% FBS and 80 µg/ml of polybrene (hexadimethrine bromide) and added to Clu177 cells that were prior seeded at 5 × 10^4^ cells per well in a 6-well plate. 48 h after transduction, the cells were analyzed using a confocal microscope to test the expression of the mt-Keima. Clu177-mt-Keima were seeded at 1 × 10^5^ cells on a 35 mm cover glass in cells were pretreated with DMEM supplemented with 2% FBS and penicillin-streptomycin for 24 h, and incubated with BSA (vehicle), PA, BSA + ONX-0914 or PA + ONX-0914 for 24 h. Before the experiment, the cell medium was replaced with DMEM Hepes 25 mM. Cells were imaged using a Leica SP8 confocal microscope and a 63X oil immersion 1.4 ma lens in an environmental chamber maintained at 37 °C. Fluorescence of mt-Keima was imaged in two channels via two sequential excitations (440 nm, cyan; 586 nm, red) and using a 620 nm emission range. The images were analyzed using ImageJ software experiments [19]. The number of acid puncta per cell, representing the number of mitochondria undergoing degradation (mitophagy), was quantified in 80 cells from 3 independent.

### Preparation of protein extracts and Western blot analysis

Cells were washed with PBS and lysed with RIPA buffer supplemented with a cocktail of protease and phosphatase inhibitors (1 mM NaF, 0.3 mM Na_2_P_2_O_7_, and 1 mM Na_3_VO_4_; Sigma-Aldrich). Cells were collected and lysed for 30 min at 4 °C. The extracts were centrifuged for 20 min at 12,000 × g at 4 °C. The supernatants were collected, and protein concentration was quantified using the BCA assay (ThermoFisher Scientific). The extracted proteins were denatured at 70 °C for 5 min. For the gels, 30–50 µg of denatured proteins from each sample were resolved on 7.5%, 10%, or 12% SDS-PAGE gels. Gels were transferred to nitrocellulose membranes and incubated with 5% BSA (BM-0150, Winkler, RM, Chile) in tris-buffered saline (TBS) to block nonspecific binding, with agitation for 1 h. The membranes were incubated with primary antibodies p62/SQSTM1, p-AKT (Ser473), AKT, BECN1, FIP200, PTEN, β5, β5i, β-actin, prepared in 1X TBS − 5% BSA overnight. Then, the membranes were washed 3 times for 10 min in TBS-0.25% Tween-20 and revealed with the appropriate horseradish peroxidase-labeled secondary antibodies (Goat Anti-Mouse IgG (H + L)-HRP Conjugate, #1,706,516; Goat Anti-Rabbit IgG (H + L)-HRP Conjugate, #1,706,515; Bio-Rad, CA, USA) and the chemiluminescent substrate.

### Native Western blot

Hypothalamus from mice fed a CHOW or HFD diet for 8 weeks were frozen and stored at -80 °C, then resuspended in 100 µL of TSDG lysis buffer (Tris/HCl 10 mM, MgCl_2_ 1.1 mM, NaCl 10 mM, EDTA 0.1 mM, NaN_3_ 1 mM, DTT 1 mM, ATP 2 mM, Glycerol 10% (v/v), H_2_O). The tissue was ground with a plastic douncer, and subsequently, it was subjected to 5 cycles of freezing/thawing in liquid nitrogen, followed by centrifugation at 20,000 x g for 20 min at 4 °C. The supernatant was taken, and after quantification, the native buffer was added. Samples were loaded into pre-made gels of NativePAGE™ 4 to 16%, Bis-Tris, 1.0 mm (Cat# BN1002BOX, Thermo Fisher Scientific), and a running buffer containing TBE buffer 1x (Tris Base 712 mM, boric acid 712 mM, EDTA 10 mM, H_2_O), ATP 413 µM, MgCl_2_ 2 mM, DTT 0.5 mM, and H_2_O. The electrophoresis chamber was placed on ice, and the electrophoresis was run at 150 V for 4.5 h. Afterward, the gel was incubated in a buffer containing the peptide that detects chymotrypsin activity (Tris pH 7.5 0.5 µM, ATP/MgCl_2_, DTT 1 mM, AC-ANW-AMC 50 µM, H_2_O) for 30 min at 37 °C, protected from light, and then visualized under UV light. To remove the residual activity buffer, it was washed with a buffer containing SDS 2% w/v, Na_2_CO_3_ 66 mM, β-mercaptoethanol 1.5% v/v, and H_2_O for 15 min at room temperature. For transfer, a buffer of 10% tris-glycine and 10% methanol in H_2_O was used, and the PVDF membrane was activated in methanol for 5 min. It was then transferred on ice in the cold room for 1.5 h at 400 mA. Afterward, it was blocked in 1X PBS 5% milk for 1 h and incubated overnight with the primary antibody β5i or RPN11. Subsequently, washes were performed with PBS Tween, incubated with the secondary antibody for 1 h, and washed with PBS 0.25% Tween-20 until visualization on the iBright™ CL1500 Imaging System (Cat#A44114, ThermoFisher Scientific Inc).

### Intracellular glucose assay

The Amplex RED kit (Cat#A22189, Thermofisher) was used to measure glucose levels in CLU177 cells treated with BSA or PA concentration for 24 h in the presence or absence of the ONX-0914 inhibitor. The cells were trypsinized, centrifuged at 800 x g for 5 min, and re-plated to keep them suspended. After being trypsinized for 30 min, insulin at 10 nM was added for 5–15 min, or 1X PBS as a control. The samples were then centrifuged at 800 x g for 5 min, and the supernatant was removed and subsequently washed with 1X PBS. The samples were centrifuged again, the supernatant was removed and then resuspended in 100 µL of 1X PBS. The samples were then sonicated for 3 s at 30 mA in the sonicator. Subsequently, 50 µL of the sample collected in 1X PBS was added to 50 µL of the Amplex RED kit in black 96-well plates. It was allowed to react for 30 min at room temperature and measured on a fluorimeter (Biotek Synergy HTX) at EX530/EM590. The values were normalized based on the protein amount quantified with the BCA kit.

### Flow Cytometry measurements

Cells were treated with BSA or PA at the indicated concentration for 24 h in the presence or absence of the inhibitor ONX-0914. After this time, cells were incubated with 1 µM of TMRE probe (Cat# T669, ThermoFisher Inc) or 5 µM of Mitosox-Red probe (Cat# M36008, ThermoFisher Inc) in combination with 0.5 µM of Mitotracker Deep Red probe (Cat# M46753, ThermoFisher Inc), all diluted in HBSS medium for 30 min. After two washes with PBS 1X, the cells were trypsinized, washed again in PBS 1X, and finally resuspended in 200 µL of HBSS for analysis in the cytometer (BD FACSCanto II).

### Statistical analysis

Densitometric quantification of the Western blot signal was estimated using Fiji version 2.1.0 (http://imagej.net/software/Fiji/). Experiments were performed at least three times for each assay. Data analysis was conducted using Microsoft Excel 2021 (Microsoft Corporation) and Prism 9.0 (GraphPad Software, San Diego, CA, USA). The graphs are presented as points ± median (U-Mann-Whitney) or mean ± standard error of the mean (SEM, for ANOVA-ONE-WAY), and comparisons were made using the non-parametric U Mann-Whitney t-test or ANOVA-ONE-WAY. Values of *p < 0.05, **p < 0.01, ***p < 0.001, and ****p < 0.0001 were considered statistically significant.

## Results

### HFD increases the levels and activity of the immunoproteasome in the hypothalamus

Mice lacking the β5i subunit of the immunoproteasome and fed with HFD for 2 months accumulate less fat and improve glucose intolerance and insulin sensitivity than wild-type mice fed with the same diet [14]. Importantly, this improvement in the metabolic phenotype occurs despite no difference in food intake, energy expenditure, or locomotor activity. Furthermore, these mice have decreased macrophage infiltration and chemokine expression, reducing inflammation in the adipose tissue compared to HFD-fed WT mice [14].

In this study, we examined how HFD impacts immunoproteasome levels in the hypothalamus of male mice compared to age-matched mice fed a regular chow diet. We focus on the β5i subunit because it is the one that would be mainly altered in obesity [14, 20]. Furthermore, this subunit is involved in the differentiation of preadipocytes to adipocytes [21] and it has been also related to lipodystrophy diseases [22]. Our findings indicate that the HFD feeding significantly increases the levels of the β5i subunit of the immunoproteasome in the hypothalamus, compared to mice fed a chow diet (Fig. 1A and B). No changes were observed in the constitutive proteasome β5 subunit (Fig. 1A and C). These results indicate that HFD feeding specifically triggers the immunoproteasome, which may contribute to the low-grade chronic inflammation associated with obesity.

We analyzed the immunoproteasome complex levels in the hypothalamus of mice fed with an HFD or chow using protein extracts resolved on native gels. We used immunodetection to analyze the 19 S and 20 S complexes, focusing on the RPN11 (19 S) and β5i (20 S) subunits. By detecting the overlapping signal of both antibodies coupled to a secondary antibody, we identified the 26 S and 30 S proteasome complexes. These complexes are formed by the interaction of a single regulatory particle 19 S with the 20 S proteasome to create the 26 S proteasome or by binding two regulatory particles 19 S with the 20 S proteasome to form the 30 S proteasome. We observed that HFD feeding significantly increases the 20 S complex in the hypothalamus of mice fed with an HFD compared to mice fed with the chow diet (Fig. 1D-E). However, we did not observe significant differences in the amount of the 26 S and 30 S complexes (Fig. 1D, F, D and G). The activity of the β5i subunit of the immunoproteasome was also measured in native gels using the fluorescent substrate Ac-ANW-AMC. This assay allowed us to visualize the 26 S and 30 S complexes (Fig. 1H). An increase in the activity of the 30 S complex of the immunoproteasome was detected (Fig. 1J) without changes in the activity of the 26 S complex (Fig. 1I). These experiments showed that consumption of an HFD increases the levels of the β5i subunit and 20 S complex in the hypothalamus and boosts the activity of the 30 S complex. These results suggest that the immunoproteasome may have a role in HFD-induced hypothalamic inflammation.

### HFD increases immunoproteasome levels in astrocytes and POMC neurons in the hypothalamus

**Fig. 2:**
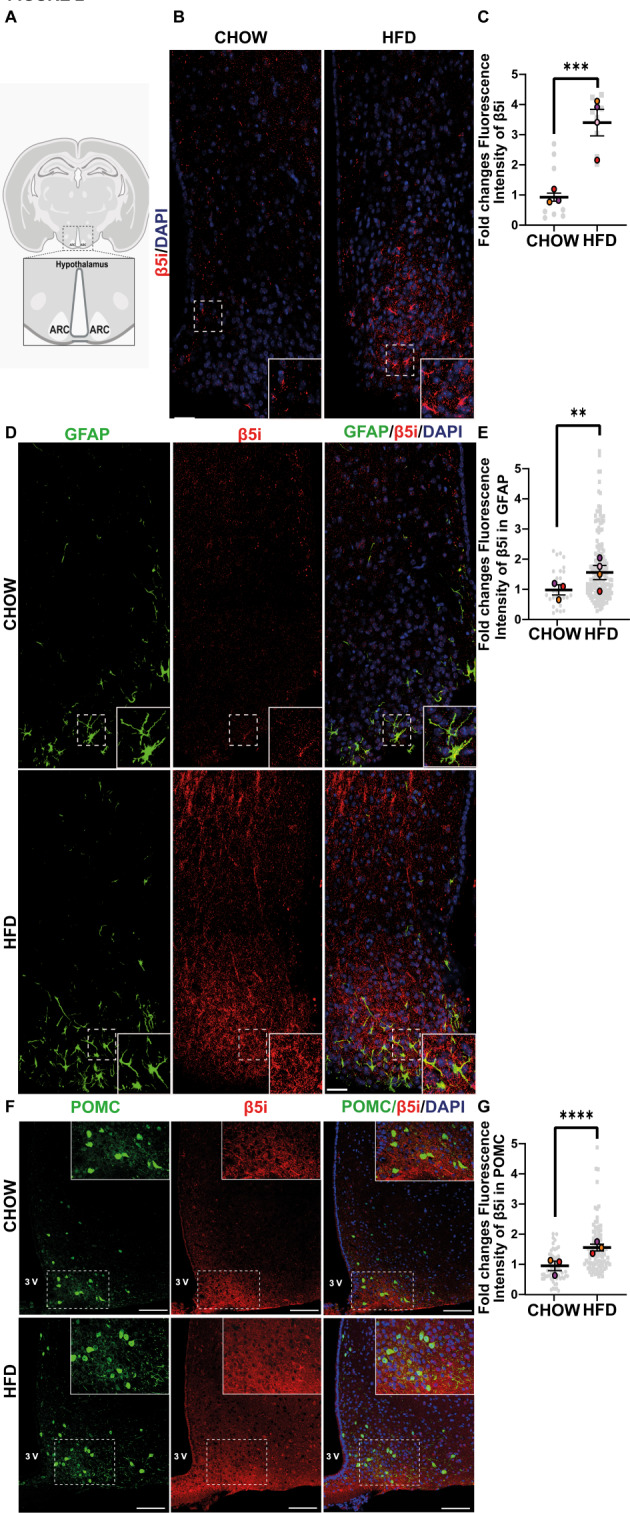
HFD feeding increases the levels and activity of the immunoproteasome in the hypothalamus (**A**) Schematic representation of a coronal section of the brain showing the location of the arcuate nucleus, where the confocal images of mice fed with chow or HFD were obtained. (**B**) Immunofluorescence using anti-β5i antibodies (red) and DAPI as a nuclei marker (blue). (**C**) Quantification of total fluorescence of the β5i subunit in all parenchyma of the hypothalamus. (**D**) Co-immunostaining using anti-β5i antibodies (red, left panel), anti-GFAP (green, middle panel), and merge image (right panel), including nuclear staining with DAPI (blue). (**E**) Quantification of total fluorescence of the β5i subunit in segmented GFAP-positive astrocytes. (**F**) Immunostaining using anti-β5i (red, left panel), POMC-GFP (green, middle panel), and composite image (right panel), including nuclear staining with DAPI (blue) Scale bar: 50 μm. (**G**) Quantification of percentage of POMC neurons segmented to B5i neurons. (A, C, E) shows a 4X magnification of the area indicated in the white dashed-line box projected in the lower-right inset. Scale bar: 15 μm. Data are represented as mean fluorescence intensity/cell ± SEM. Analysis: U-Mann-Whitney test. *p < 0.05, **p < 0.01, ***p < 0.001, ****p < 0.0001. *n* = 4

To analyze the distribution and levels of the immunoproteasome, we performed immunofluorescence staining of the β5i subunit of the immunoproteasome on the ARC (arcuate nucleus), obtained from mice fed with an HFD or chow diet. We observed an increase in the total fluorescence intensity of the immunoproteasome in the ARC, close to the 3 V, and in the median eminence (Fig. 2B-C). According to the location, shape, and immunopositivity of GFAP, some correspond to astrocytes (Fig. 2D-E). However, the increase in total fluorescence of β5i is probably attributable to other parenchymal cells like neurons or microglia. Therefore, we explore the localization of immunoproteasome in POMC-GFP mice exposed to either diet. We observed a significant increase in the number of POMC neurons positive to β5i in mice fed with a HFD compared to CHOW (Fig. 2F-G). On the other hand, we observed that the HFD feeding also increases the immunoproteasome fluorescence intensity in different brain areas, such as the motor cortex M1 and M2, where suggestive labeling of pyramidal neurons is observed (Fig. S1 A-B). This suggests that the effects of HFD feeding on brain might affect global cerebral homeostasis, impacting extrahypothalamic regions.

### The inhibition of the immunoproteasome with ONX-0914 restores the imbalance of the insulin-glucose axis induced by PA in the presence of insulin

**Fig. 3:**
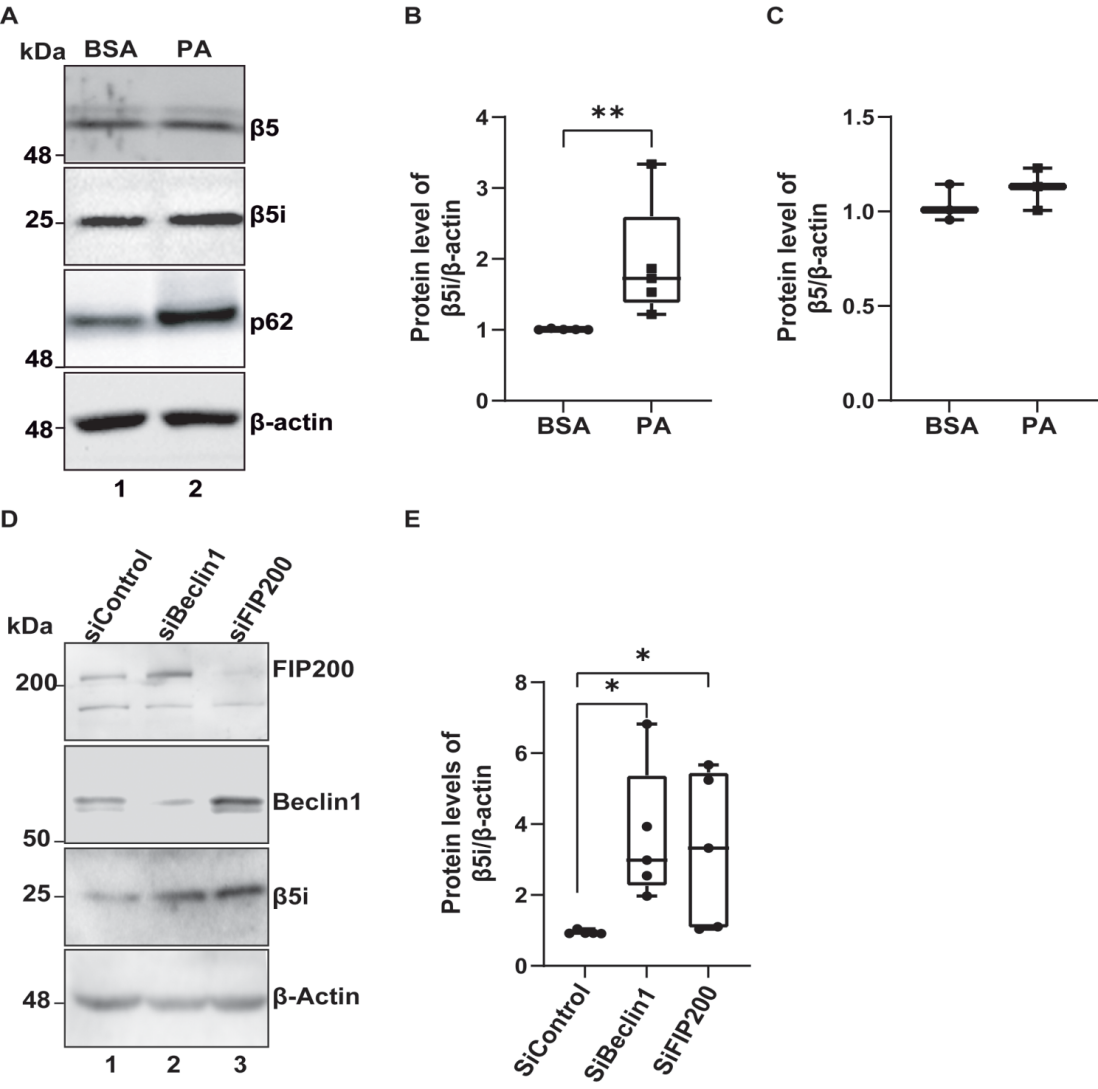
Inhibition of autophagy increases the β5i subunit of the immunoproteasome in CLU177 hypothalamic neuronal cells (**A**) CLU177 cells were incubated with vehicle (BSA) or PA (100 µM) for 24 h. Protein extracts were analyzed by western blot to assess the levels of the β5i subunit of the immunoproteasome, β5 subunit of the proteasome, and p62/SQSTM1. (**B**) Quantification of β5i subunit levels normalized to the internal control β-actin. (**C**) Quantification of β5 subunit levels normalized to the internal control β-actin. (**D**) CLU177 cells were transfected for 72 h with liposomes containing specific siRNA sequences targeting Beclin1 and FIP200, including a siControl sequence. Extracts were analyzed by western blot to assess the levels of the β5i subunit of the immunoproteasome and the silenced proteins FIP200 and Beclin1. (**E**) Quantification of β5i subunit levels in CLU177 relative to the internal control β-actin with the indicated treatments. Data are represented as mean ± SEM. Analysis: U-Mann-Whitney test. *p < 0.05. B and D (*n* = 5) and C (*n* = 3)

It has been observed that the process of autophagy is not functioning properly in the hypothalamus of male mice who have been fed an HFD for a prolonged period of time [5, 6, 23]. Additionally, an increase in saturated fatty acids such as PA has been found in the brains of mice fed with an HFD for 16 weeks [4, 24, 25]. Consistently, it has also been observed that the PA reduces the autophagic flux in N43/5 cells, a model of embryonic hypothalamic neurons [7]. We observed that in CLU177 cells, a model of adult hypothalamic neurons [26], treatment with PA for 24 h increases the β5i subunit of the immunoproteasome but not the β5 subunit of the proteasome (Fig. 3A-C). This result confirms that saturated fatty acids, enriched in the HFD diet, upregulate the immunoproteasome. This increase in the immunoproteasome could be partly due to a loss of autophagy since the silencing of the essential autophagy-related genes Beclin1 and FIP200, both involved in the formation of the autophagosome [27], augment immunoproteasome levels (Fig. 3D-E, Fig. S2).

Given that autophagy inhibition blunts insulin signaling in hypothalamic neurons [7] and that consumption of HFDs causes hypothalamic insulin resistance in rodents [17], we studied whether the increased in immunoproteasome upon PA treatment (Fig. 3D-E) could play a role in the insulin pathway. To this aim, we used ONX-0914, a specific inhibitor of the immunoproteasome [11]. First standardized the concentration of the immunoproteasome inhibitor ONX-0914 to evaluate its effect on the insulin pathway (Fig. S3).

**Fig. 4:**
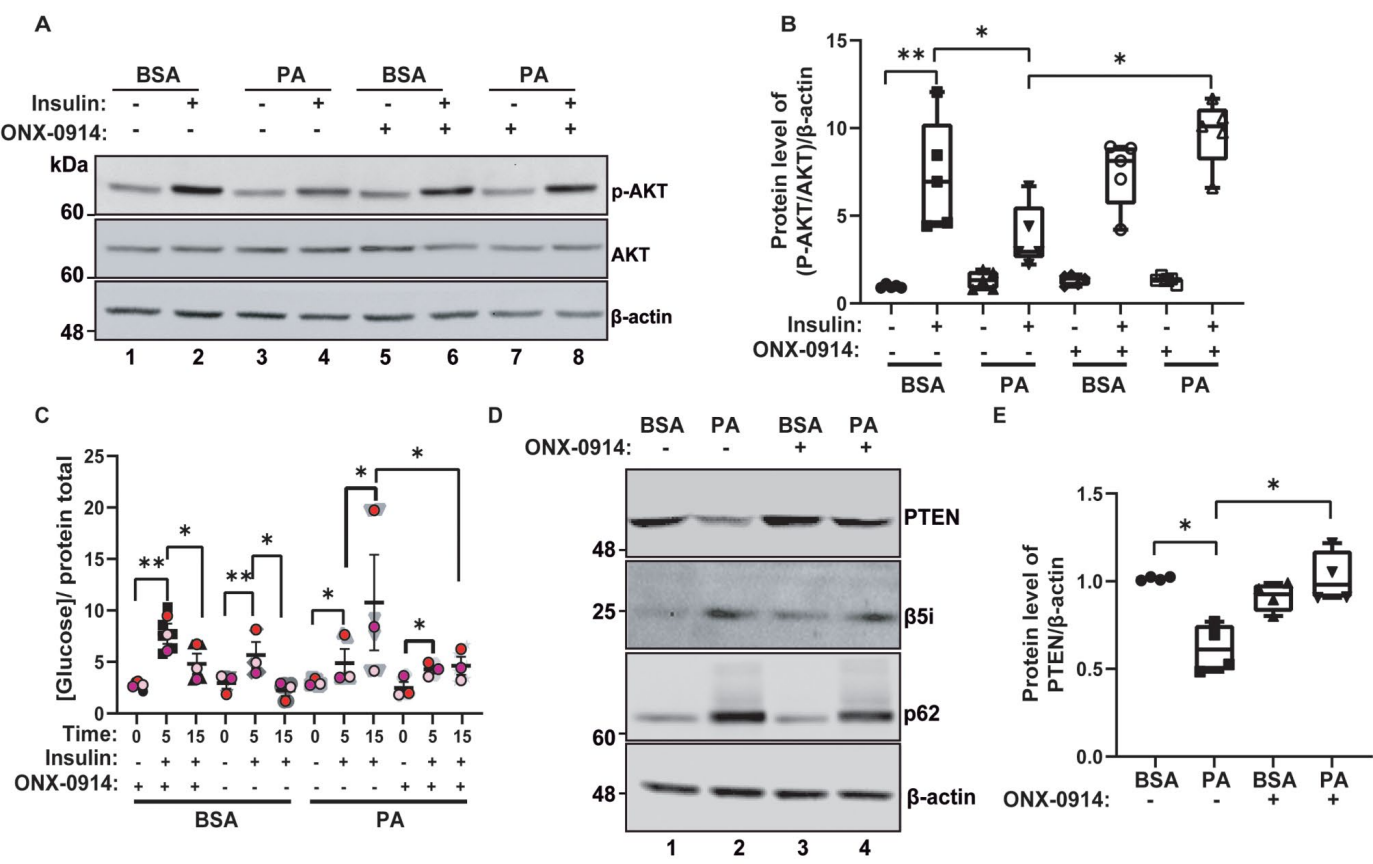
ONX-0914 attenuates the PA-induced reduction in PTEN levels and insulin sensitivity in CLU177 (**A**) CLU177 cells were incubated with vehicle (BSA) or PA (100 µM) for 24 h in the presence or absence of 100 nM ONX-0914. Protein extracts were analyzed by western blot to evaluate the levels of p-AKT(Ser-473) and total AKT. (**B**) Quantification of the P-AKT/AKT(S-473) ratio normalized to the internal control β-actin. (**C**) Intracellular glucose levels measured by AmpliRed Glucose/Glucose Oxidase assay in CLU177 cells treated with vehicle (BSA) or PA (100 µM) for 24 h in the presence or absence of ONX-0914, followed by stimulation with 10 nM insulin or PBS 1X (vehicle) for 5 or 15-min. (**D**) Protein extracts were analyzed by western blot to assess the levels of the β5i subunit of the immunoproteasome, PTEN, and p62/SQSTM1. (**E**) Quantification of PTEN levels normalized to the internal control β-actin. Data are represented as mean ± SEM. Analysis: U-Mann-Whitney test. *p < 0.05. (B and E) *n* = 5 and (**C**) *n* = 3.

CLU177 cells were treated with PA for 24 h, in the absence and presence of ONX-0914. As expected, insulin treatment increased the phosphorylation of AKT(S-473) in the BSA condition, while the addition of PA along with insulin decreased its phosphorylation, a phenomenon previously documented [7]. Interestingly, the ONX-0914 inhibitor rescued the AKT(S-473) phosphorylation levels in the presence of PA (Fig. 4A-B). To further understand the relationship between insulin signaling, the immunoproteasome, and glucose, we analyzed intracellular glucose levels in CLU177 cells after insulin treatment. To this purpose, we evaluated intracellular glucose levels using a kit with glucose oxidase enzyme. We studied glucose levels after 5 and 15 min of exposure to insulin. Insulin stimulation was performed in cells previously incubated with vehicle (BSA) or PA (100 µM) for 24 h, both in the absence and presence of the ONX-0914 inhibitor. In the conditions with the BSA vehicle and the absence of ONX-0914, we observed that insulin caused a significant time-dependent increase in intracellular glucose levels. Specifically, we observed an increase in glucose levels after 5 min of insulin, which was not evident after 15 min of insulin (Fig. 4C). A similar result was obtained in the presence of the BSA vehicle and the ONX-0914 inhibitor (Fig. 4C). However, unexpectedly, we observed a different pattern when the cells were incubated with PA. In this case, intracellular glucose levels continued to increase at 15 min of insulin incubation (Fig. 4C), indicating a dysregulation due to the effect of PA. Interestingly, the increase in glucose at 15 min by PA was reduced in the presence of ONX-0914, approaching the control condition with BSA (Fig. 4C).

It has been shown that PTEN, a negative regulator of the insulin pathway, is a substrate of the immunoproteasome [28]. We observed that PTEN levels decreased after 24 h of treatment with PA, accompanied by an increase in the b5 subunit of the immunoproteasome and p62/SQSTM1, an autophagy marker (Fig. 4D-E). We observed that the reduction of PTEN, induced by PA, was attenuated in the presence of ONX-0914 (Fig. 4D-E), suggesting that the decrease in PTEN by PA could be partly dependent on the immunoproteasome, as previously described in UO2S cells [29]. Interestingly, the ONX-0914 inhibitor did not cause significant changes in the levels of the β5i subunit of the immunoproteasome under any of the tested conditions (Fig. 4A, C). Additionally, although the increase in p62/SQSTM1 levels, a characteristic phenomenon of PA treatment due to autophagic flux inhibition, did not show significant changes in the presence of ONX-0914, there was a subtle inclination towards a decrease. These results suggested that the inhibition of the immunoproteasome and the inflammation caused by PA could have a preventive effect on the imbalance of the insulin-glucose axis. This effect could be partly related to the restoration of PTEN levels.

### ONX-0914 restores mitochondrial membrane potential, decreasing ROS and mitophagy enhanced by PA treatment

**Fig. 5:**
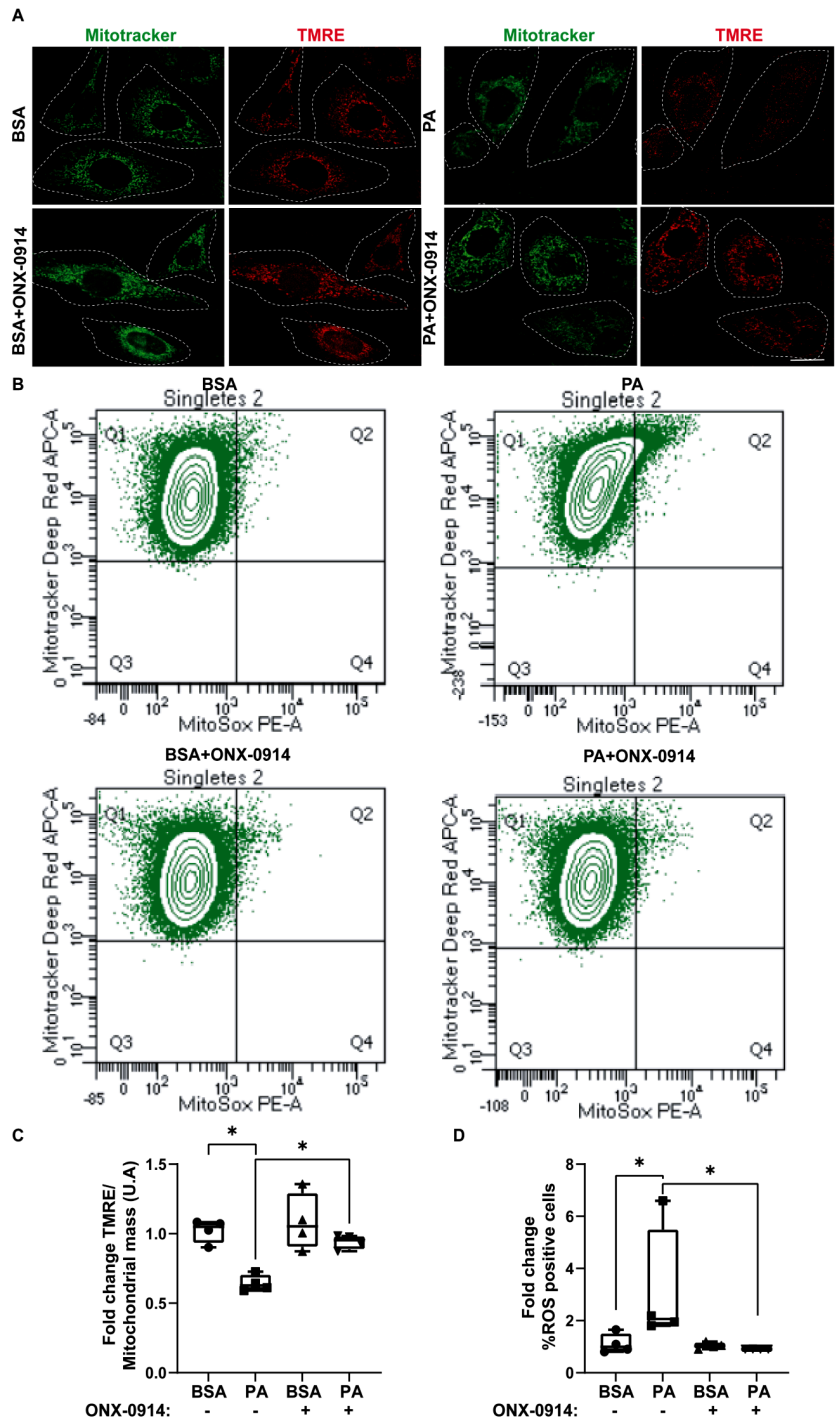
ONX-0914 reverses the decrease in mitochondrial membrane potential and increase in ROS induced by PA (**A**) Representative image of mitochondrial membrane potential labeled with TMRE probe (red) and mitochondria labeled with Mitotracker probe (green) in CLU177 cells treated with BSA or PA (100 µM) in the presence or absence of ONX-0914 for 24 h. (**B**) Flow cytometry to detect changes in Mitosox-Red for ROS measurement together with Mitotracker-Deep Red for mitochondrial mass measurement. (**C**) Quantification of TMRE fluorescence intensity normalized to the amount of Mitotracker via flow cytometry. (**D**) Quantification of Mitosox fluorescence intensity (ROS) via flow cytometry. Data are represented as mean ± SEM. Scale bar = 10 μm. Analysis: U-Mann-Whitney test. *p < 0.05. *n* = 4

We considered the implications of the Randle cycle in line with the idea that PA might influence cellular metabolism and consequently alter how cells handle the use of glucose following its entry [30]. This cycle proposes a metabolic competition between glucose and free fatty acids, considering that there should always be a balance between glycolysis and mitochondrial β-oxidation [30]. Under high concentrations of fatty acids and mitochondrial β-oxidation conditions, glycolysis should be inhibited, a mechanism that would occur due to high citrate production in the mitochondria. This metabolite has been shown to inhibit key enzymes of glycolysis resulting in intracellular glucose accumulation [30, 31]. Mitochondrial membrane potential is crucial for maintaining the physiological function of the respiratory chain and generating ATP, while reactive oxygen species (ROS) are signaling molecules that lead to oxidative and cellular stress [32]. Therefore, we assessed the integrity and activity of mitochondria in the presence of PA in CLU177 cells. Initially, we evaluated mitochondrial function through ROS and membrane potential marked with Tetramethylrhodamine ethyl ester perchlorate (TMRE), aiming to assess whether mitochondria in the PA + ONX-0914 condition could show functional improvements. We observed that PA causes a decrease in mitochondrial membrane potential, manifested by a reduction in TMRE fluorescence intensity (Fig. 5A, C). Additionally, PA increases Mitosox fluorescence intensity, which measures ROS levels (Fig. 5B, D). However, both the decrease in mitochondrial membrane potential [33, 34] and the increase in ROS [35, 36] have been documented in other cell types.

Surprisingly, we observed that the ONX-0914 inhibitor reduces the effects of PA on mitochondrial function, revealing an unexpected role of the immunoproteasome in this regulation.

Next, we proceeded to analyze whether PA caused changes at the level of the mitochondrial network in CLU177 cells compared to the BSA condition. For this purpose, we analyzed mitochondria using the protein TOM20, located in the outer membrane of mitochondria (Fig. S4). These results indicated that PA generates a less complex mitochondrial network with less branched and interconnected mitochondria, suggesting a more fragmented mitochondrial network (Fig. 4S). This confirms previous studies with PA in other cell types [34, 37, 38]. Unexpectedly, when we tested the combined effect of PA with ONX-0914, we observed even greater mitochondrial network fragmentation. Since ONX-0914 enhances mitochondrial function in combination with PA, the increased mitochondrial fragmentation could positively impact mitochondrial activity (Fig. S4).

Mitochondrial dynamics play a crucial role in responding to mitochondrial dysfunction by promoting the elimination of irreversibly damaged mitochondria through mitochondrial fission [39]. In this regard, mitophagy is a protective mechanism that removes dysfunctional mitochondria while preserving the population of healthy mitochondria. It has been observed that PA plays a dual role in endothelial cells [40]. Depending on the stress level, adding PA triggers mitophagy activation to remove damaged mitochondria. However, under severe stress, this mechanism can be inhibited, leading to the accumulation of damaged mitochondria [40].

**Fig. 6:**
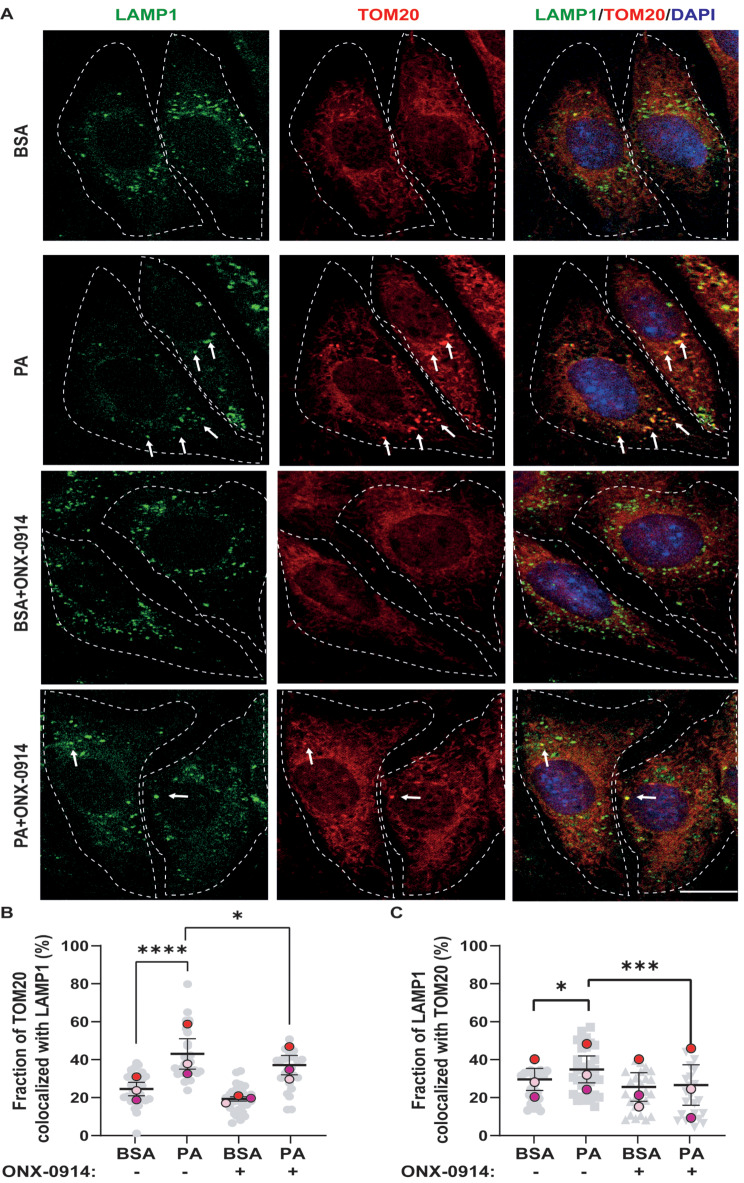
ONX-0914 reduces the co-localization of TOM20 with LAMP1 increased by PA treatment (**A**) Representative confocal image of CLU177 cells treated with BSA or PA (100 µM) in the presence or absence of ONX-0914 for 24 h and co-stained with anti-TOM20 antibodies (Red, left panel), anti-LAMP1 antibodies (Green, middle panel), and composite image with DAPI (Blue, right panel). (**B**) % co-localization between TOM20 and LAMP1 according to Manders’ coefficient analysis. (**C**) % co-localization between LAMP1 and TOM20 according to Manders’ coefficient analysis. Scale bar = 10 μm. Data are represented as mean ± SEM. Analysis: ONE-WAY ANOVA test. *p < 0.05, ***p < 0.001, ****p < 0.0001. *n* = 3

Based on these findings, we aimed to evaluate mitophagy in our model. For this purpose, we studied the co-localization of LAMP1, an endolysosomal marker, and TOM20, an outer mitochondrial membrane marker. This would allow us to assess whether PA, by blocking autophagic flux, could prevent the degradation of damaged mitochondria, potentially resulting in co-localization between both markers. Thus, we observed that PA caused a significant increase in the co-localization of TOM20 and LAMP1 compared to BSA. This suggested that PA, by blocking autophagic flux in the presence of dysfunctional mitochondria, could inhibit the final stage of mitophagy (Fig. 6A-C). Interestingly, we observed that ONX-0914, in the presence of PA, where the mitochondria are even smaller, reduced the co-localization between TOM20 and LAMP1 (Fig. 6A-C).

Since we observed a more significant co-localization of LAMP1 with TOM20 in the PA condition compared to the control, we conducted experiments to confirm if this was related to mitophagy.

To differentiate free mitochondria from mitochondria within lysosomes (mito-lysosomes), we used the pH-sensitive mt-Keima reporter. This tool has dual excitation peaks in its protonated and deprotonated states, making it a ratiometric and reversible pH measure.

**Fig. 7:**
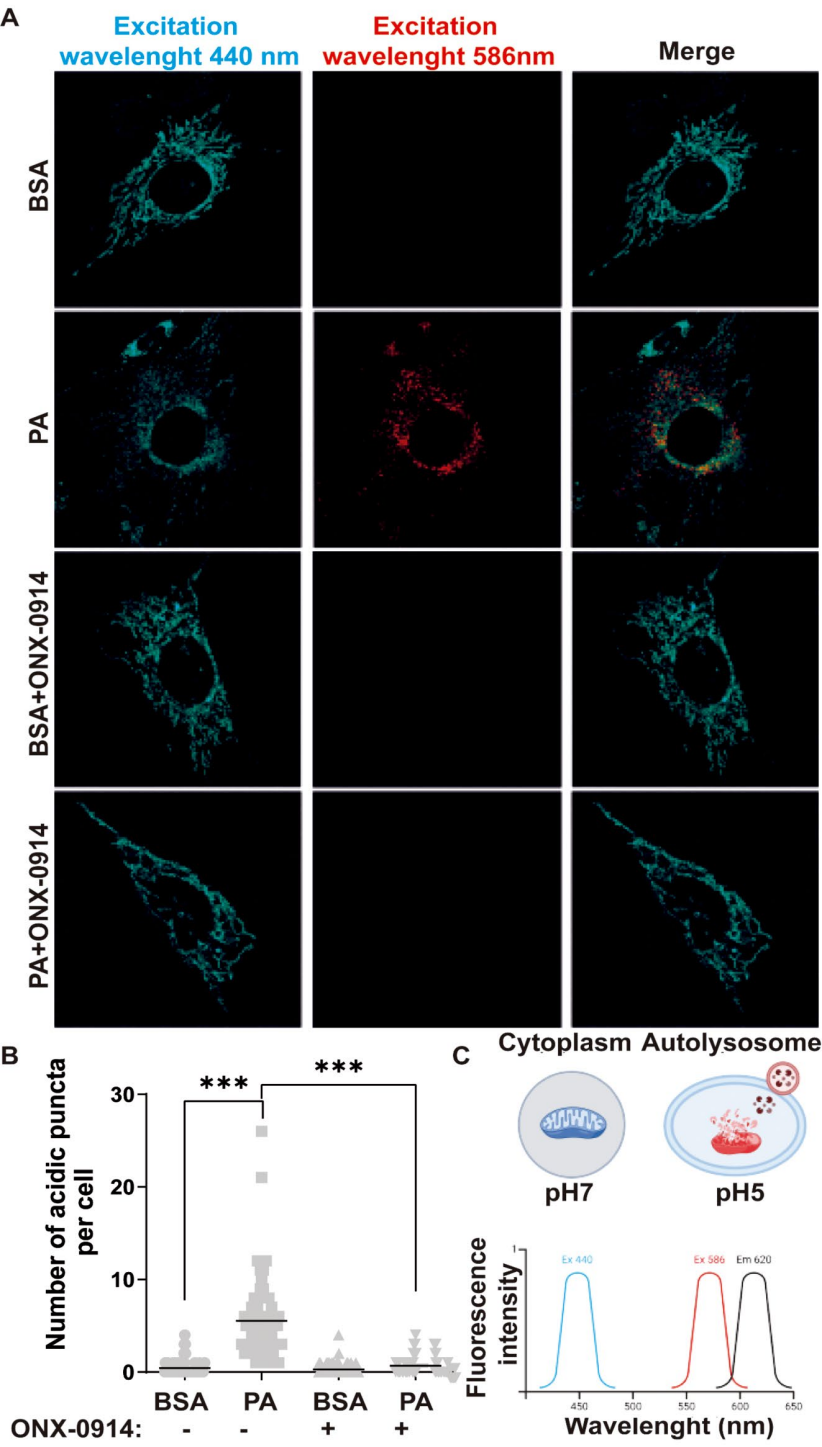
ONX-0914 Reverses Mitophagy Induced by PA treatment (**A**) Representative confocal images of CLU177 cells treated with BSA or PA in the presence or absence of ONX-0914 for 24 h and transfected with mt-Keima were excited by the 440 and 586 nm laser. (**B**) Quantification of the number of acid puncta per cell (**C**) Schematic depiction of the ratiometric mitophagy reporter mt-Keima. Scale bar = 10 μm. Data are represented as mean ± SEM. Analysis: ONE-WAY ANOVA test. *p < 0.05, ***p < 0.001, ****p < 0.0001. *n* = 3

In the presence of BSA, we observed a significant emission at 440 nm (Fig. 7A-B). Conversely, when PA was used, an emission at 586 nm was detected (Fig. 7A-B). These findings confirm PA caused accumulation of damaged mitochondria in lysosomes through mitophagy. Further, we found ONX-0914 caused a decrease in the emission at 586 nm compared to PA alone (Fig. 7.A-B), consistently with the idea that this inhibitor prevents mitochondrial damage, and therefore degradation, by PA. Together, these results confirm the pathogenic role of the immunoproteasome in response to PA in hypothalamic neurons, an effect related to the unexpected regulatory role of the immunoproteasome on mitochondrial function.

## Discussion

Our results demonstrated that the β5i subunit of the immunoproteasome is increased in proteins and activity levels in the hypothalamus of mice consuming HFD in comparison to those on a chow diet. Furthermore, using native western blotting, we observed an increase in the assembly of the 20 S proteasome complex and a rise in the 30 S proteasome complex (19–20 S-19 S), coupled with a significant increase in its activity. Although the activity of the 30 S complex is higher in the hypothalamus of this mouse model of diet-induced obesity, the implications of these findings on the hypothalamic neuroendocrine integrative function, as well as the effect on other body systems that contribute to the function of this important area of the brain remain to be characterized [41]. We suggest that this increase may correlate with a tendency towards higher protein levels of the 30 S complex in the hypothalamus of HFD-induced obese mice, suggesting that the immunoproteasome is elevated and active in the hypothalamus of obese mice. Several reports have shown that proinflammatory cytokines or pathological conditions can increase the immunoproteasome in neurons, astrocytes, and microglia [42–45]. Our results suggest this could also be the case in the context of obesity induced by HFD. Additionally, we describe for the first time that the immunoproteasome is also present in POMC neurons, where it is increased following HFD consumption, suggesting a potential role in neuroinflammation. However, immunoproteasome expression on the function of these and other hypothalamic neurons remains to be elucidated.

On the other hand, previous studies have shown that PA reduces AKT phosphorylation levels [7], which have been associated with insulin resistance in different models [46–48]. Consistently, we observed a decrease in AKT phosphorylation after 24 h of PA treatment, which was restored when the immunoproteasome was chemically inhibited by treatment with ONX-0914. We observed that insulin stimulation for 5 min increases intracellular glucose levels in both control conditions and in the presence of PA, with or without the ONX-0914 inhibitor. However, after 15 min of insulin stimulation, we observed that in control conditions, intracellular glucose levels decrease, suggesting that, after glucose uptake, this fuel is rapidly utilized. In contrast, with PA, we observed a sustained increase in intracellular glucose, a phenotype that is rescued in the presence of the ONX-0914 inhibitor. There is evidence indicating that PA treatment decreases glucose consumption [7, 49, 50], while other studies report the opposite [51, 52]. This suggests that the effects of PA on glucose levels vary depending on the time, concentration, and cell type. A potential explanation for this phenomenon is the metabolic pathway glucose undergoes upon entering the cells. For example, it has been shown that PA affects the activity of hexokinase, the first enzyme in glycolysis [53–55]. It is possible that PA could affect glycolysis, upregulating fatty acid oxidation to increase energy production or other pathways, such as the acetylation of STAT3, which enhance phospholipid biosynthesis, thereby increasing mitochondrial membrane lipid content to resist apoptosis. as previously reported [56]. Therefore, studying various metabolic pathways and different cell types is essential to gain a deeper understanding of the effects of PA on cell metabolism. To investigate this, it is necessary to thoroughly examine the various metabolites that are enhanced in response to PA overload using metabolomics and other omics techniques. This approach could provide a broader view of metabolism under the overload of saturated fats or upon the overexpression of the immunoproteasome, an aspect not addressed in this study.

Another possibility is that PA itself could exert an effect on GLUT4, GLUT1, or the main glucose transporter localized in neurons GLUT3 on the cell surface of hypothalamic neurons, which might explain the increase in intracellular glucose, a hypothesis that requires future investigation. Nonetheless, it is still possible that a reduction in PTEN levels by PA could at least partially mediate these effects.

It has been described that the immunoproteasome degrades PTEN, a lipid and protein phosphatase sensitive to reduction-oxidation, playing an essential role in regulating the PI3K-AKT pathway [28]. PTEN often exhibits genetic alterations in cancer, such as point mutations, chromosomal deletions, or epigenetic mechanisms. Furthermore, it is also dysregulated in PIP3 metabolism, an effect observed in diabetes [57]. It is important to note that PTEN activity is regulated by post-translational modifications, such as phosphorylation or oxidation, which affect its activity and interaction with other proteins. The relationship between obesity and PTEN is still a subject of discussion and controversy in the scientific community. However, it has been consistently observed that PTEN loss is associated with improved insulin sensitivity in obesity models [58–60]. However, a decrease in PTEN levels may also promote fat storage due to the increased efficiency of the PI3K-AKT pathway, limiting the organism’s ability to burn excess calories. It is essential to consider that decreasing PTEN levels can also increase the proliferation of cancer cells in an obesity context [61].

In our study, we observed that PTEN levels were significantly decreased following 24 h-PA exposure. Similar findings were recently published in U2OS cells [29]. The decrease in PTEN suggests a disruption in the PI3K-AKT pathway. Although PTEN activity can be measured, we did not assess it in this study. Instead, we used the decrease in its levels as an indicator of its dysfunction. This limitation highlights the need for future studies to directly measure PTEN activity to confirm the impact of PA in the axis PI3K-AKT/PTEN pathway. We observed that the reduction of PTEN levels, caused by 24 h of PA treatment, was reversed when using the immunoproteasome inhibitor ONX-0914. This finding suggests that the immunoproteasome plays a major role in metabolism, controlling the entry of nutrients such as glucose and PA, and regulating the capacity of hypothalamic neurons to orchestrate systemic metabolism to either burn or store excess nutrients. In this regard, the immunoproteasome can emerge as a promising therapeutic target with potential impact in the treatment of metabolic diseases. Although our results strongly support this hypothesis, further in vivo studies with the ONX-0914 inhibitor are needed to confirm it.

Additionally, we used the protein p62/SQSTM1 to demonstrate that PA inhibits the autophagic flux; nonetheless immunoproteasome inhibition is insufficient to restore autophagy basal levels in the cells used in this study. However, we do not rule out that immunoproteasome inhibition could account for a distinct function of p62/SQSTM1. p62/SQSTM1 is modified and regulated by phosphorylation, ubiquitination, acetylation, proteolytic processing, and the formation of disulfide bridges, where these modifications fine-tune p62/SQSTM1 activities in autophagy [62, 63]. On the other hand, it is essential to note that we would expect that a decrease in PTEN levels would increase the PI3K-AKT pathway and, therefore, enhance AKT phosphorylation. Nonetheless, it has been described that a reduction in PTEN levels can activate the ERK pathway, and ERK activation can inhibit AKT phosphorylation, which could explain our findings [64, 65]. Therefore, we attribute the decrease in AKT phosphorylation to the recovery of PTEN levels due to immunoproteasome inhibition.

Dysfunction in glucose metabolism could also indicate alterations in fatty acid metabolism and essential organelles that control these pathways. The interaction between glucose and fatty acid metabolism, known as the Randle cycle or glucose-fatty acid cycle, is critical to understanding processes that may be linked to the insulin-glucose imbalance. This raised the possibility that excess of PA and the immunoproteasome could affect glucose metabolism. In this regard, the functional relationship between the immunoproteasome and mitochondria is related to the fact that PA promotes mitochondrial fragmentation. This phenomenon has been described in many cell types and observed in CLU177, a cellular model of adult hypothalamic neurons. Our results showed that ONX-0914, in combination with PA, promotes even greater mitochondrial fragmentation than PA alone. In this regard, it is known that mitochondrial fission is often responsible for mitochondrial fragmentation, favoring the metabolism of exogenous fatty acids and the transfer of fatty acids between mitochondria and lipid droplets [37]. Therefore, a possibility suggested by our results is that the inhibition of the immunoproteasome by ONX-0914 could favor mitochondrial fission to reduce the excess of PA and its lipotoxicity. However, mitochondrial fragmentation can also promote the elimination of dysfunctional mitochondria through mitophagy. This option is supported by (i) the fact that while PA leads to a loss of mitochondrial membrane potential and an increase in ROS, these alterations are not observed in the presence of ONX-0914, (ii) the colocalization of TOM20 with LAMP-1 in the presence of PA is lost with ONX-0914 and (iii) mt-Keima shows mitochondria in acid compartment in the presence of PA, an effect that is reversed by ONX-0914 exposure. This strongly suggests that inhibiting the immunoproteasome prevents mitochondrial damage in the presence of PA, allowing mitochondria to be more active in carrying out PA β-oxidation. How could the immunoproteasome affect mitochondrial integrity? We observed that ONX-0914 reduces mitochondrial damage in the presence of PA, as demonstrated by a reduction in ROS, an increase in membrane potential, and the absence of mitophagy. It is well-known that the proteasome plays a crucial role in mitochondrial protein quality control [66]. Mitochondria-associated degradation (MAD) acts on two levels: firstly, the proteasome removes mature, functionally compromised, or mis-localized proteins from the mitochondrial surface; secondly, the proteasome clears import intermediates that are stalled during translocation from the mitochondrial import pore [54]. However, little is known about how the immunoproteasome regulates mitochondrial function. In this regard, it has been observed that mitochondrial damage or dysfunction leads to increased immunoproteasome levels, which has been linked to contributing to autoimmunity [67]. On the other hand, it has been observed that the induction of the subunit β1i of the immunoproteasome is required to regulate cell proteostasis upon mitochondrial dysfunction [68] or subunit β1i deficiency causes abnormal metabolism, oxidative stress, and neuroinflammation [69]. In contrast, the β2i subunit of the immunoproteasome ameliorates myocardial ischemia/reperfusion injury by regulating Parkin-Mfn1/2-mediated mitochondrial fusion [70]. As for the effect of the β5i subunit of the immunoproteasome on mitochondria, it is unknown. A report using the inhibitor ONX-0912, which inhibits the β5i subunit, showed that it triggered apoptosis through the intrinsic mitochondrial pathway and induced mitophagy by activating the Parkin/Pink pathway in liver cancer [71]. In contrast, another report indicates that inhibition of chymotrypsin-like activity of the proteasome by Ixazomib prevents mitochondrial dysfunction during myocardial ischemia by blocking the release of cytochrome c and significantly preserving mitofusin-2 integrity [72].

This disparity in these results could indicate that it depends on the cell type and the cellular context. Based on our results, the effect of the immunoproteasome on mitochondrial function could be related to PTEN. PTEN has two isoforms: PTENα (PTEN-L) and PTENβ; PTENα promotes the recruitment of Parkin, an E3 ubiquitin ligase, to damaged mitochondria, while PTENβ can prevent Parkin from recognizing and tagging damaged mitochondria for degradation [73]. This dual effect allows the maintenance of mitochondrial integrity. In our case, we observed a decrease in total PTEN levels. However, it is likely that the PTENα isoform is the one that decreases the most and accounts for this effect, so future research could delve into this mechanism.

Therefore, another possibility is that the immunoproteasome inhibits the autophagic flux. In this regard, it has been recently reported that the immunoproteasome degrades the autophagy-related proteins ATG5 and ATG12 in cardiomyocyte and retinopathy models [74, 75], something that could be occurring in the context of hypothalamic neurons. Our results suggest that PA elevates the immunoproteasome due to autophagy inhibition. This protein complex could further enhance the inhibition of autophagy by degrading proteins involved in this pathway. This would explain the rescue effect of ONX-0914 on mitochondrial function by favoring mitophagy and improvements in LAMP-1 positive compartments in the presence of PA. Additionally, the immunoproteasome would impact the insulin-glucose axis by reducing PTEN levels, resulting in increased intracellular glucose. This phenotype could also be a consequence of excessive β-oxidation, supported by mitochondrial fragmentation in the presence of PA, an effect that could be partially compensated by efficient mitophagy.

Our results show an increase in the immunoproteasome in POMC neurons. These neurons induce satiety and respond to adipogenic signals such as insulin. Thus, we propose that the immunoproteasome is a molecular pathogenic factor in hypothalamic neurons within the context of HFD-induced obesity and that its pharmacological inhibition may offer a promising therapeutic strategy to prevent the onset of metabolic alterations associated with the development of obesity-related metabolic diseases, such as insulin resistance. Additionally, we observed an increase in the immunoproteasome in astrocytes. Other authors have already documented that the immunoproteasome increases in astrocytes and microglia, a product of inflammation [42, 76]. Therefore, it was expected to observe this increase. However, it remains to be determined if this increase can be harmful, as in the case of neurons. Our findings support the hypothesis that the immunoproteasome may play a crucial role in the hypothalamic neuroinflammation caused by HFD and obesity. We also underscore the role of the immunoproteasome in controlling mitochondrial homeostasis, revealing a new and unexpected mechanism involved in regulating the insulin/glucose balance in hypothalamic neurons. However, it is still necessary to investigate how this mitochondrial dysfunction induced by the immunoprpteosome impacts the function of these neurons and the impact on the neuroendocrine integration commanded by these neurons to maintain body energy homeostasis.

Altogether, our findings reveal the unexpected role of the immunoproteasome in cell metabolism and obesity, highlighting the pathogenic role of hypothalamic low-grade inflammation and the necessity of maintaining intracellular integrity to prevent energy imbalances caused by key actors in proteostasis and inflammation. While further studies are needed to validate this hypothesis and understand the impact of immunoproteasome inhibition on metabolic imbalances related to hypothalamic function and obesity, it is crucial to address the limitations associated with immune system inhibition by ONX-0914, which affects antigen presentation required for virus and tumor cell elimination [11]. Additionally, a thorough characterization is necessary to determine whether ONX-0914 can cross the blood-brain barrier and be delivered to the brain, particularly in the context of obesity [77]. Our findings underscore the significant role of hypothalamic organelle integrity and the potential of immunoproteasome inhibition as a therapeutic approach for preventing metabolic and inflammatory diseases.

### Electronic supplementary material

Below is the link to the electronic supplementary material.

Supplementary Material 1

Supplementary Material 2

Supplementary Material 3

Supplementary Material 4

## Data Availability

Data is provided within the manuscript or supplementary information files.

## Abbreviations

APS: Ammonium persulfate
ARC: arcuate nucleus
CHOW: Regular diet
FBS: Fetal bovine serum
HFD: High Fat Diet
MAD: Mitochondria-associated degradation
OPG: retroviral gag/pol
PA: Palmitic Acid
PEI: Polyethyleneimine
PFA: Paraformaldehyde
ROS: Reactive oxygen species
SA: Stearic Acid
TMRE: Tetramethylrhodamine ethyl ester perchlorate
VSV-G: Plasmids viral capsid
