# Supplementary figures and images for: Targeting the immunoproteasome in hypothalamic neurons as a novel therapeutic strategy for high-fat diet-induced obesity and metabolic dysregulation
Source: J Neuroinflammation. 2024 Aug 2;21:191. doi: 10.1186/s12974-024-03154-z (PMC11297766; doi:10.1186/s12974-024-03154-z)

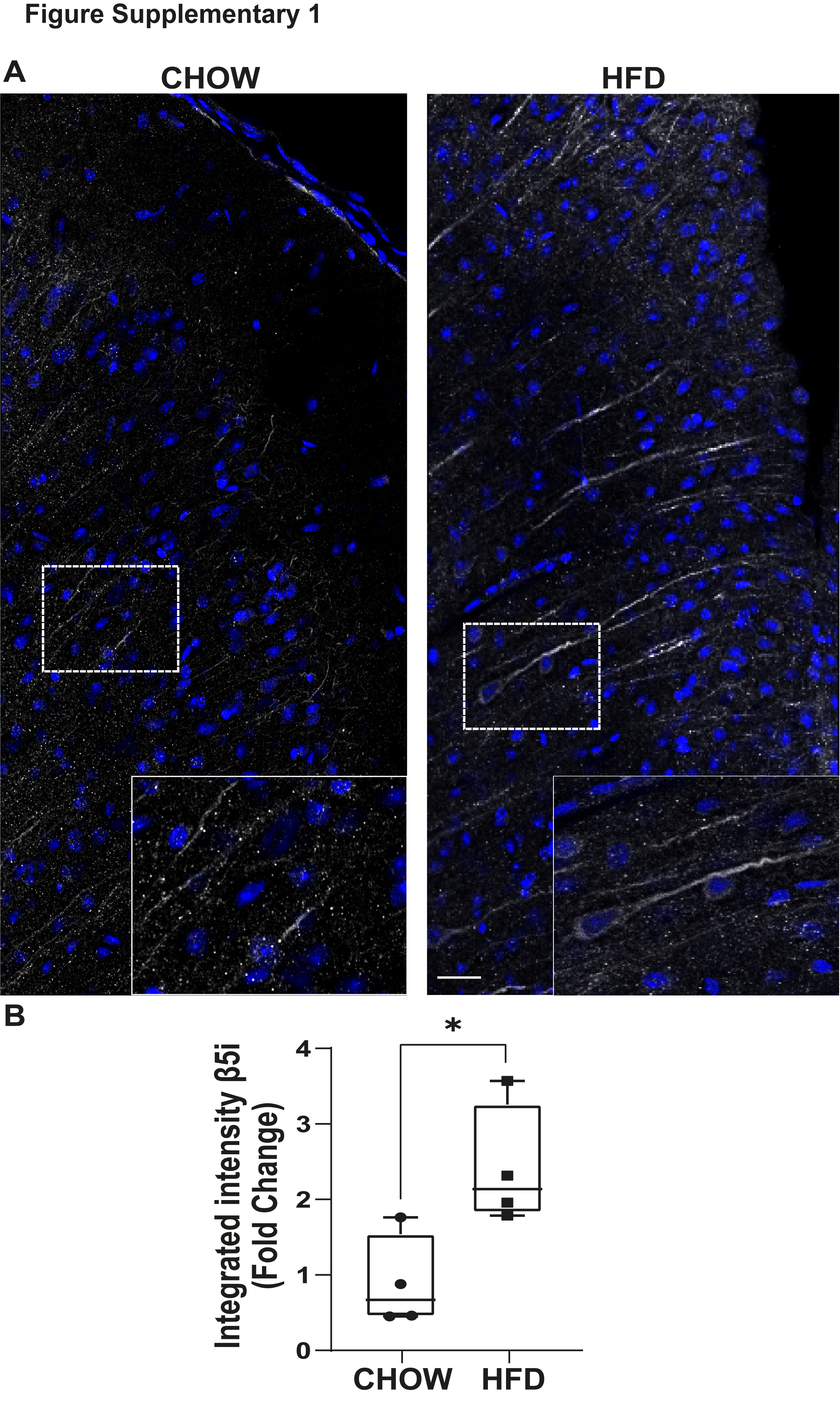

Supplement: Supplementary file 1 — Supplementary Material 1 [file 12974_2024_3154_MOESM1_ESM.tif]

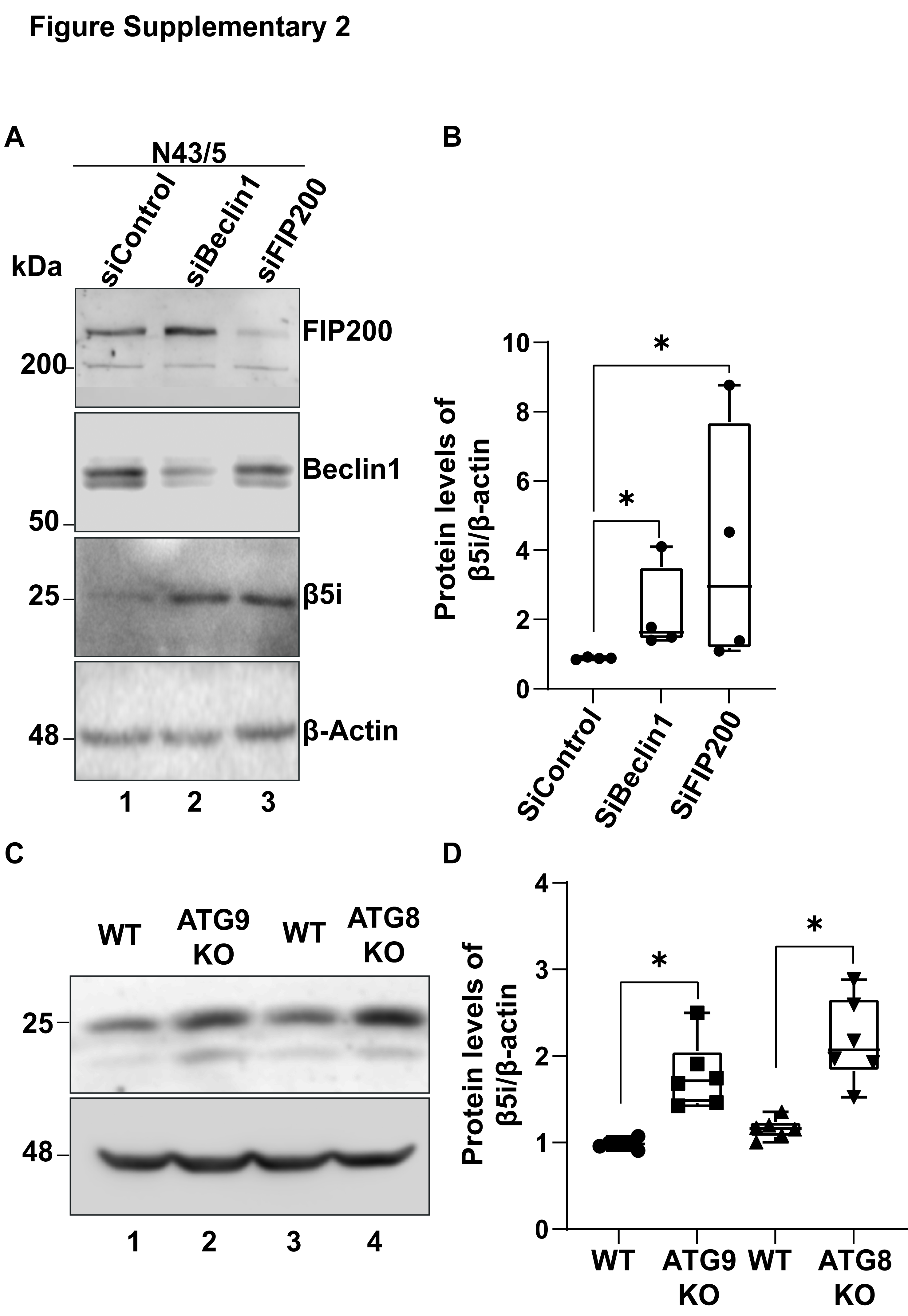

Supplement: Supplementary file 2 — Supplementary Material 2 [file 12974_2024_3154_MOESM2_ESM.tif]

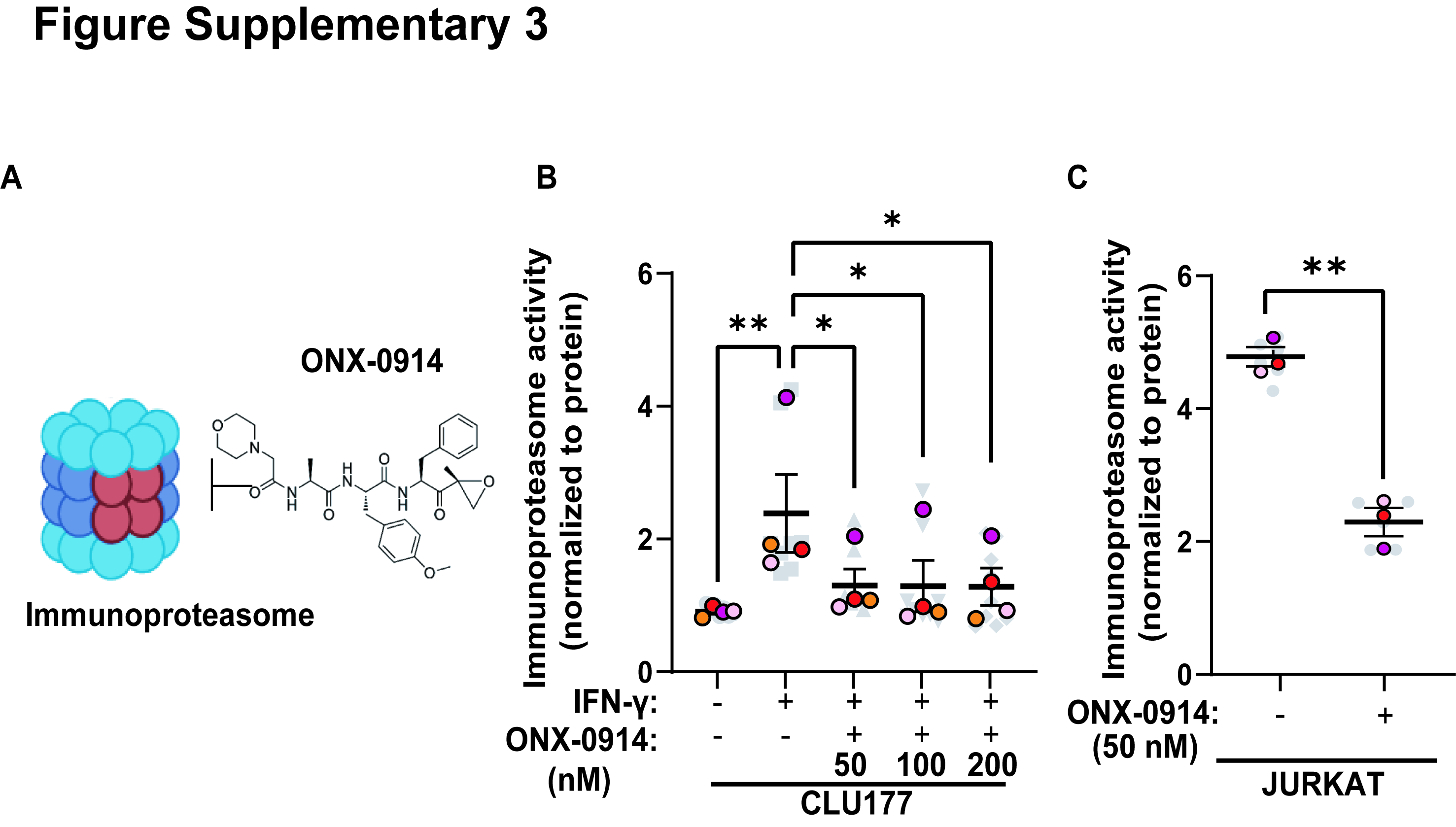

Supplement: Supplementary file 3 — Supplementary Material 3 [file 12974_2024_3154_MOESM3_ESM.tif]

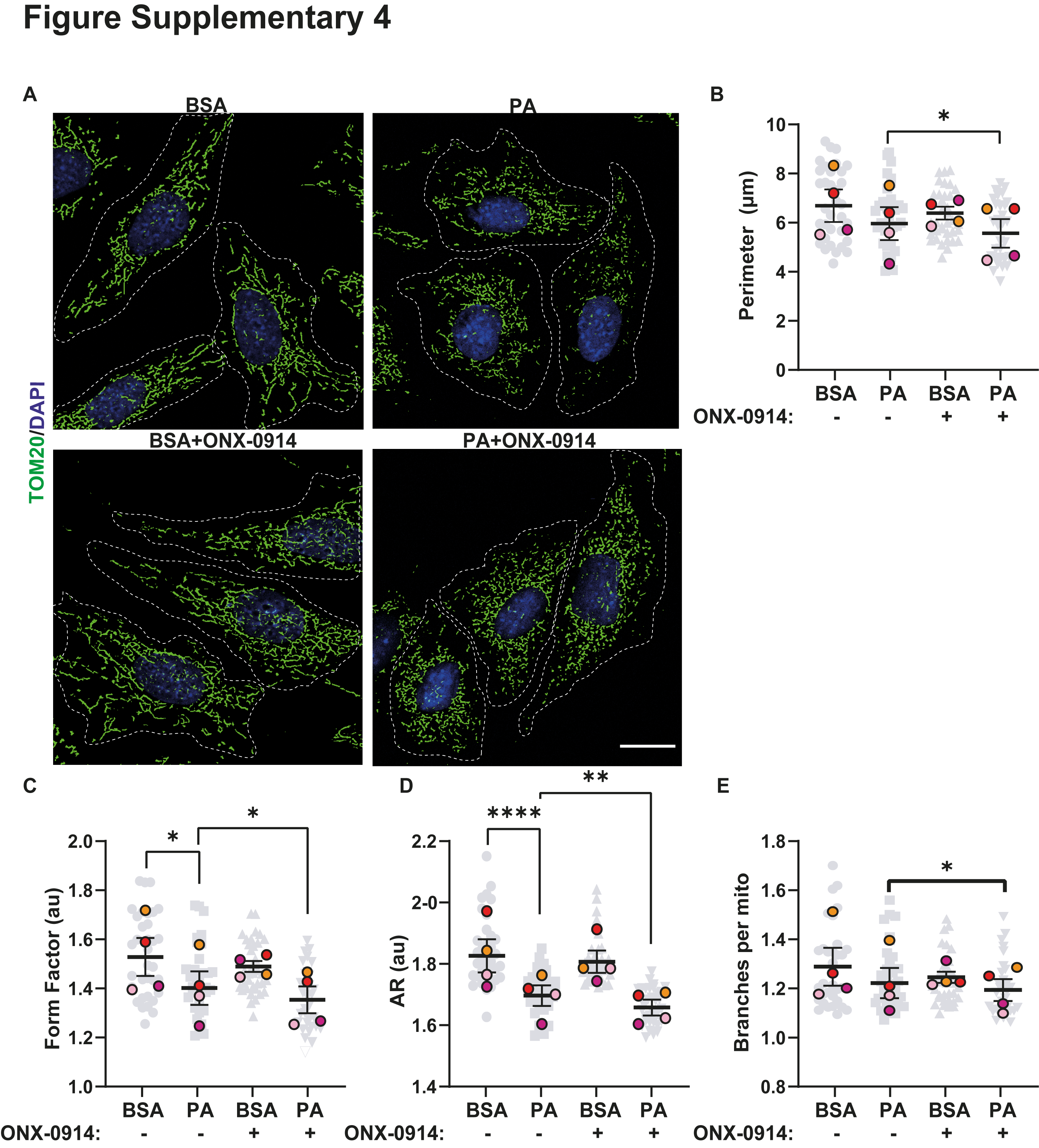

Supplement: Supplementary file 4 — Supplementary Material 4 [file 12974_2024_3154_MOESM4_ESM.tif]
